# Sero-Diagnosis of Paternity

**Published:** 1913-09

**Authors:** 


Sero-diagnosis of Paternity. Mayoral and Jimines, *La. Med. Scientif.*, Feb., 1912, following the analogy of Eksberg's specific cancer reaction, claim that emulsions of erythrocytes in isotonic NaCl solution, injected under the skin of pregnant women produce an erythematous spot within six hours, if the erythrocytes are derived from the man by whom they are pregnant, otherwise not. They have controled the test in various ways and claim that it is reliable. Various allusions to this report have appeared in American journals, but we have seen no recent articles confirming or discrediting the claim.

